# Mechanical and Fire Performance of Innovative Hollow Glue-Laminated Timber Beams

**DOI:** 10.3390/polym14163381

**Published:** 2022-08-18

**Authors:** Nikola Perković, Vlatka Rajčić

**Affiliations:** Structural Department, Faculty of Civil Engineering, University of Zagreb, 10000 Zagreb, Croatia

**Keywords:** timber, laminated, innovative, hollow, fire, adhesive, fiber-reinforced, intumescent, paint

## Abstract

Fire safety greatly contributes to feeling safe, and it is a key parameter for the selection of building materials. The combustibility of timber is one of the main reasons to have the strict restriction on timber for use as a building material, especially for multistory buildings. Therefore, the main prerequisite for the use of timber in buildings is to ensure adequate fire resistance, using passive and active fire protection measures. This article contains the results of mechanical and fire experimental tests of both normal and innovative hollow glued laminated timber beams. A total of 10 timber beams were tested at ambient temperature, and 3 timber beams in fire conditions, which differed in cross-section type but also in the applied fire protection. The first beam was a normal GL beam without fire protection, the second a hollow beam covered by intumescent paint, while the third was also hollow, additionally protected by mineral wool infill inside the holes. The load-carrying capacity of the hollow beam in ambient conditions was estimated at 65% of the load-carrying capacity of a normal GL beam. Fire tests indicated that hollow timber beams with both intumescent paint and mineral wool infill failed at a similar time as a normal GL beam without fire protection. One-dimensional β_0_ and notional charring rates β_n_ were obtained. Time to the protective material failure was 17 min. The main cause of failure of hollow beams was the appearance of delamination due to the reduction of the lamella bonding surface.

## 1. Introduction

Recently, the design and construction of mass-produced buildings using cross-laminated timber (CLT) and glued laminated timber (GLT) has risen sharply. Among other reasons, this is because timber is the only material that absorbs carbon dioxide. Furthermore, timber construction satisfies architectural aspirations, and may ultimately result in reduced costs and higher construction speeds, compared to standard, commonly non-combustible materials, and structures. At the end of the life cycle, timber releases stored carbon into the atmosphere by decomposition or combustion. Therefore, the main prerequisite for the use of timber in buildings is to ensure adequate fire resistance, using passive and active fire protection measures. Among other things, passive protection systems include various boards and coatings. One way is the impregnation of wood with fire retardants or coating with fire-resistant coatings. Although numerous coatings provide significant fire protection for wood, they are sensitive to mechanical damage, so numerous approaches to fully impregnating wood with fire retardants have been studied [1]. The challenge of developing a suitable fire retardant lies in the many requirements of its properties. It must be durable, i.e., there must be no deterioration in durability or mechanical properties, it must not be toxic and contribute to the generation of smoke, and it must be resistant to washing off when used on external surfaces. Some types of fire retardants prevent combustion by releasing inert gas that decomposes the previously flammable gas mixture; that is, the concentration of oxygen in the zone of contact with wood, which is required for combustion, decreases. On the other hand, there are those fire retardants that promote the formation of a charred layer, which is a natural way of protecting wood from fire because it reduces the penetration of oxygen and heat into the interior, i.e., into the area of non-charred wood. The influence of fire retardants has been investigated by Chu et al. in their two related papers [2,3]. This work investigated the fire resistance and compression recovery properties of poplar treated with a functionalized surface layer, formed by a novel combined treatment of nitrogen–phosphorus fire retardant (NP) pre-impregnation and thermomechanical densification (TM). The heat release rate of the combined treated wood was 48.1% lower than that of untreated wood, and the CO yield was 68.4% lower than that of only NP-treated wood. Moreover, the compression recovery in the thickness was 77.2% lower than that of solely TM-treated wood [2]. Following on from this research, the influence of dynamic wettability and bonding strength has also been shown [3]. The low compression stability of the TM-only treated poplar causes surface swelling during the wetting process and decreases the bonding strength. Compared with that of wood that was only heat-treated, the surface bonding strength of the NP–TM combined treated poplar increased by 53.3% [3].

The reason for the significant consequences of a fire is the two main products of fire action: high temperatures and large amounts of smoke. High temperatures will affect the structure itself, i.e., its load-bearing capacity and safety, while smoke will affect people by reducing their visibility when finding a way out and making it difficult for them to breathe. The building can be considered to be fire resistant if is able to meet the required load-bearing capacity (R) and/or thermal insulation (I) and/or other expected attribute during the fire event. Furthermore, each material/product contributes to the development of fire due to its own decomposition, which occurs through the combustion of that substance under certain test conditions (reaction to fire). For non-combustible materials, the main parameters are weight loss and temperature rise, while the main parameters of flammable materials are ignition ease, flame spread, discharge, smoke, and heat release rate. Timber as a material and timber-based panels are classified in category D [4]. If the timber is protected by coatings that slow down the spread of fire, it can be classified in class B, but the classification of the timber element as non-combustible, i.e., class A1 or A2, cannot be achieved. Reaction to fire can be reduced by one class in the interior of a building if an automatic fire extinguishing system has been installed [5]. Additional separation of materials includes separation in the form of production fumes (s1–s3) and is also related to the fuel of material dripping in case of fire (d0–d2) [6].

### 1.1. Debonding and Delamination

An important step in the production of laminated timber elements (GLT and CLT) involves stacking and gluing pre-treated lamellas into the composition of the laminated timber. The gaps between the lamellas should be minimized to fit the requirements of the physics of the building (fire resistance, air permeability, or sound insulation), esthetics, and the technique of joining the boards. By splitting lamellas into larger parts and then rejoining them, the possibility of imperfections declines because the variance is reduced [7]. To achieve (re)connection, adhesive technology is widely used for engineering timber products (EWP) [8].

However, in many technical regulations, including EN 16,351 [9], maximum spacing of up to 6 mm only is allowed. In the case of production of a CLT element without gaps, some manufacturers first make individual layers of the CLT element by gluing the narrow edges of the lamellae, after which the layers made in this way are glued together on wide sides into the integrity of the final element. However, the contribution from the application of adhesive on the narrow sides of the lamellae is questionable and in principle, it is recommended that it can be neglected or at best limited only to the inner layers of the element. It is necessary to analyze the behavior of the structure under normal conditions, but also under higher temperature conditions. Fire safety greatly contributes to the overall feeling of safety, and it is a key parameter for the selection of building materials. Mechanical and fire loading, along with geometric and material properties of timber structures, play a decisive role in the theoretical investigation into the time-dependent fire resistance of these elements. The mechanical strength of timber is defined by many factors, such as moisture content, density, grain slope, and natural imperfections. The behavior of the EWP depends on the quality of the adhesive and the quality of the connection with the timber. As a rule, under normal circumstances, the collapse of solid timber will occur sooner than the glue itself [10]. In load-bearing constructions, adhesives must transfer high static and dynamic mechanical loads. In considering the fire events that the glue-laminated timber might be exposed to, together with a combination with other external loads, the question that arises is related to the adhesive response in such conditions; that is, the way that elevated temperatures affect its performance. Shear and normal strength are known to decrease with increasing temperatures [11]. In their work, Crielaard et al. [12] showed how delamination can be avoided by making the first lamella thicker, but another possibility was also investigated. Namely, by using the appropriate type of glue during the production of CLT elements, i.e., during the gluing of lamella layers, the delamination process can be prevented, which would avoid re-intensification of the fire, and the possibility of self-extinguishing would be realized [13].

Debonding occurs when an adhesive stops sticking (adhering) to an adherend or substrate material [14]. If this happens in the event of a fire, together with the char falling off, it ensures a new fuel load to be consumed, prolonging the decay phase and postponing the self-extinction [15]. The adhesive does not have to be organic, polymeric material; it could be an inorganic coating, for instance. Debonding occurs if the physical, chemical or mechanical forces that hold the bond together are broken, perhaps by a force or environmental attack. A particularly interesting phenomenon that negatively affects the behavior of timber in a fire event is that during delamination, the protective charcoal layers fall off and the timber remains unprotected again. In other words, the layer of self-insulation would be neglected. By evaluating the current state of the art regarding the behavior of GLT and CLT elements in a fire event, it can be concluded that the behavior of these elements during combustion can be very different from the behavior of solid timber [16]. The reason lies in the layered glued structure and the joints between the layers, which can lead to a local increase in charring [17]. Fragicomo et al. [18] showed that the burning of a CLT board is not affected by the orientation of the layers. Furthermore, it was determined that the delamination effect occurs with CLT elements. The effect of the type of glue on delamination was dealt with by Menis [19], Friquin [20], and Klippel et al. [21].

There is a general assumption that debonding and faster char fall-off are influenced by adhesive. If the char falls off at the adhesive line, a conservative criterion could be that the failure was induced by 200 °C temperature because the charred front is not expected to exist at lower temperatures. However, if delamination occurs, the temperature could be lower [22]. The next phenomenon, which is related to the previous one, but still requires a distinction to be made, is delamination. Delamination is a failure in laminated material, often a composite, which leads to the separation of the layers of reinforcement or plies. Delamination failure can be of several types, such as a fracture within the adhesive or resin, or debonding of the resin from the structural element. In the last instance, it is the debonding that leads to delamination, which helps to illustrate the distinction between debonding and delamination. Debonding is when two materials stop adhering to each other. Delamination is when a laminated material becomes separated, perhaps induced by poor processing during production, impact in service, or some other means [14].

**Figure 1:**
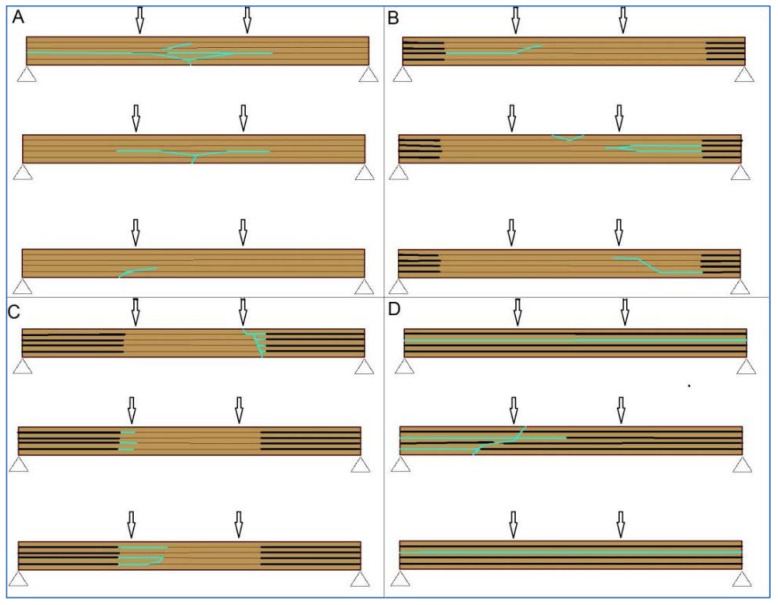
Possible failures of GL timber beam in fire event: (**A**) failure without delamination; (**B**) delamination occurs at the supports; (**C**) partially developed delamination (**D**) delamination occurs along the entire GL timber beam.

Figure 1 shows possible failures, where the blue lines represent the failure lines, the black lines represent delamination, and the dark brown lines represent the glue lines.

### 1.2. Objectives

The main goal of this research was to evaluate the behavior of innovative hollow glued laminated timber beams under ambient and fire conditions, and to compare them with standard GL timber elements. The contribution aim can be defined as follows:

- Influence of the adhesives on the load-carrying capacity of glued laminated hollow timber elements;
- Influence of elevated temperature on the adhesive and the load-carrying capacity of glued-laminated hollow timber elements;
- Impact of the perforation of the timber element on the development of temperature towards the interior of the element;
- The effect of the reduced glued surface affects the possible separation of the lamellae-delamination;
- The effect of passive fire protection on the fire resistance of hollow timber beams (one-dimensional and notional charring rate);
- Introduction of all mentioned problems in the form of the analytical or numerical model.

## 2. Materials and Methods

### 2.1. Preliminary Research—Mechanical Performance of Innovative Hollow Glue-Laminated Timber Beams

**Figure 2:**
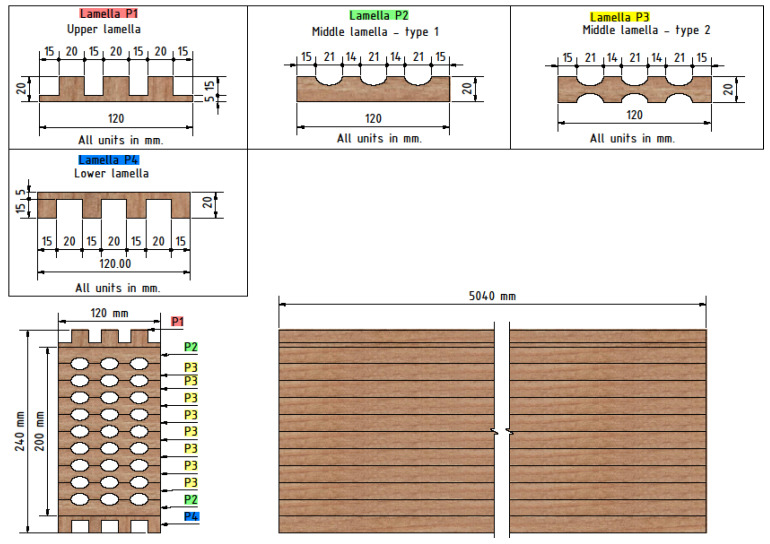
Innovative glue-laminated hollow timber beam.

This research is a continuation and improvement of investigations previously done by Perkovic et al. [23]. It concerns timber elements whose cross-section has been perforated in order to have less mass. Furthermore, this greatly facilitates the manipulation of individual elements that can be assembled into walls and ceilings. On top of all that, the remains of the perforated elements are recycled and used further for various purposes, which ensures and maintains global energy stability and reduces carbon production. The material characteristics of the samples were the same as in the previous research. The elements were made of softwood with the predominant use of European fir (Abies alba). In previous work, the basic conclusions about the behavior of these innovative elements were made, and the shortcomings and recommendations for further research were identified. Primarily, they concern the type of hole (circular or elliptical), the arrangement of the lamellae and hollows, and finally, the adhesive used in joining the lamellae. Consequently, the conclusion was that samples with elliptical holes have higher and satisfactory load capacity. Furthermore, moisture-curing 1-component polyurethane adhesive PUR Kleiberit 510.0 with reinforced fibers [24] was used for the research, and the edge lamellas were made without holes, to avoid stress concentration in the edge zones. According to DIN EN 14,080 and DIN EN 15,497, this is a one-component certified PUR adhesive for load-carrying wood construction. It is approved according to SANS 10183-2 as a wood adhesive for load-carrying wood components in service class S3 (service class S3), which means that is suitable for long-term outdoor use, even with direct contact with the ground. New, improved timber elements used in this paper can be seen in Figure 2.

**Figure 3:**
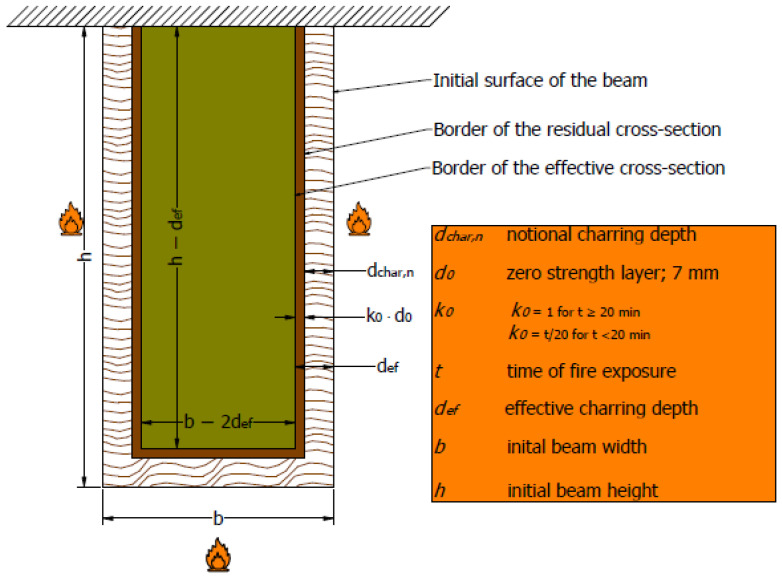
Definition of ideal residual cross-section (EN 1995-1-2, 2004 [25]).

The fire resistance time for a normal GL timber beam can be estimated by defining the ideal residual cross-section (Figure 3) according to EN 1995-1-2 [25], and comparing the actual bending stress, which depends on the residual cross-section values at a given time:

I_(y,ef)_(t) = ((h − d_ef_ (t))^3^·(b − 2d_ef_ (t))/12 (1)

σ_Ed_ = M_Ed_/I_(y,ef)_(t) · (h − d_ef_(t))/2 ≤ f_m.u,occ_ (2)

The values for β_n_, k_0_, and d_0_ are taken from Eurocode 5 (EN 1995-1-2, 2004) [25]:

β_n_ = 0.7 mm/min; k_0_ = 1; d_0_ = 7 mm (3)

The acting moment depends on the load level m_u,fi_ related to the bending moment resistance at normal temperature:

I_y,20_ = (h3 − b)/12 (4)

M_Ed_ = m_u,fi_∙(2I_y,20_ · f_m.u,occ_)/h (5)

This means the fire resistance does not depend on the bending strength but the load level factor.

m_u,fi_ ≤ (h − d_ef_(t))^2^ · (b − d_ef_(t))/(h^2^ · b) (6)

The applied load level in the fire test needs to be defined before the test. Time is the only unknown parameter remaining in the inequality, which means it can easily be computed for each load level. In this research, the load is defined respecting the serviceability limit state, i.e., the maximal allowable deflection L/300, which for this specific case is 14.4 mm. This corresponds to approximately 30% of the failure load at ambient temperature, which is the load level proposed by many researchers [26].

**Figure 4:**
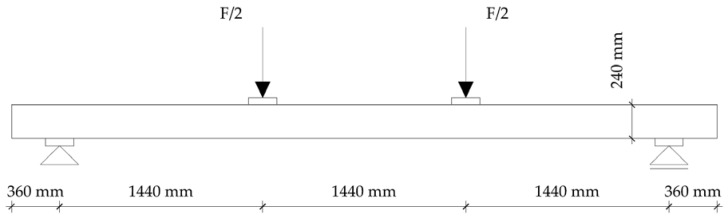
Test setup–4-point bending.

To determine the load level for the fire test, four-point bending tests on timber elements were carried out according to EN 408 [27]. The tests were carried out on a simply supported beam with a span of 18 h, where h is the height of the sample. The distance of the load input from the support and the distance between the forces themselves was 6 h (Figure 4). To avoid local stresses, additional steel plates were placed at the point of load application. In this way, the correct load distribution on the sample was obtained.

**Figure 5:**
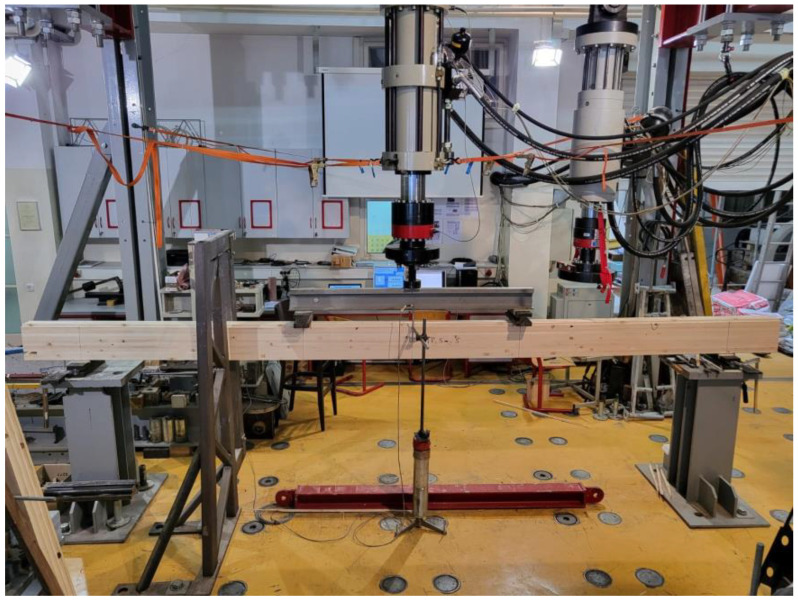
Experimental work—4-point bending at ambient temperature.

For the experimental investigation in ambient temperature, a dynamic–static universal machine from Zwick Roell GmBH Test & Co., KG, Ulm, Germany was used. The maximum force on the machine is 250 kN, class 1, which represents a deviation of 1. Furthermore, MGC plus data acquisition system (manufacturer HBM—Hottinger Baldwin Messtechnik, Germany) was used. Following EN 408 and the required failure time, the displacement rate was defined and thus the load on the sample was defined. According to EN 408, failure load should occur at (300 ± 120) s. Consequently, the test speed was defined to be 6 mm/min. In this experimental research, six basic values were measured, including time, deflection on supports and in the middle span, piston displacement, and load. Linear variable differential transformers with a sensitivity of 10 mm (supports) and 100 mm (midspan) were used to measure the displacement. Given the geometry of the sample and the fact that the end lamellae were shaped in such a way that there were notches, it was necessary to introduce additional auxiliary elements in the form of caps, over which the load was applied. For this purpose, additional timber elements were made and positioned at the load input area. These additional elements were made of a more solid material to prevent local embossing. The clamps were installed at the support in order to simulate lateral restraints. The experimental work and setup are shown in Figure 5.

**Figure 6:**
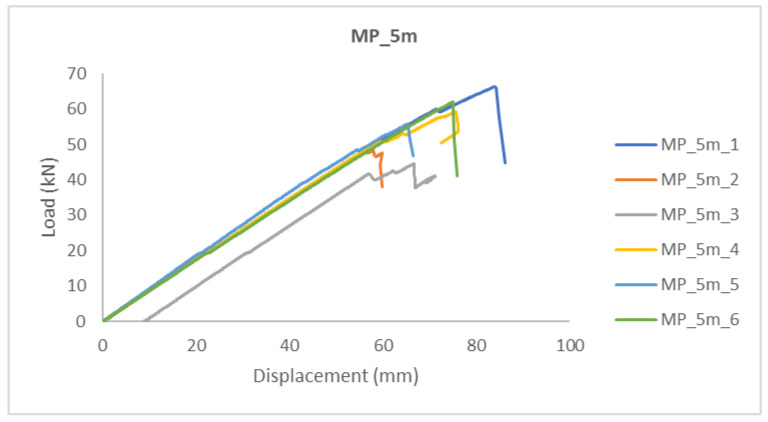
Load-displacement—normal (non-perforated) samples.

**Figure 7:**
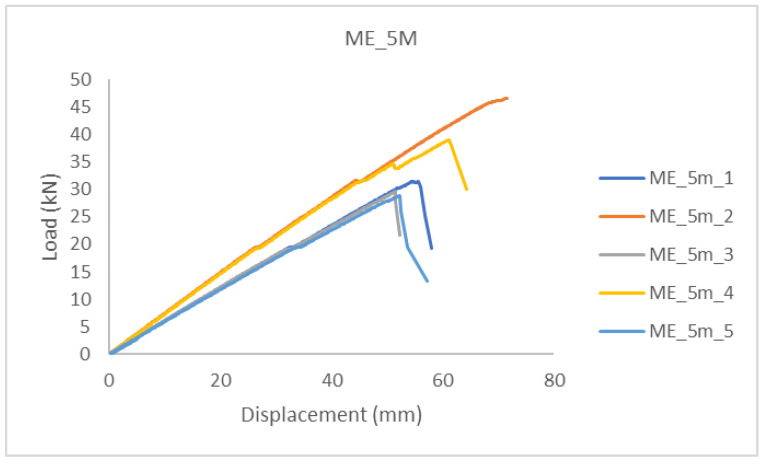
Load-displacement—hollow glued laminated samples.

In the diagrams shown below (Figure 6 and Figure 7), it can be seen that the beams (in the same sample group), up to a certain load level showed similar behavior, i.e., stiffness. For higher load levels, their stiffness was significantly reduced due to local wood imperfections, cracking, and adhesive behavior. The failure occurred after reaching the tensile strength of the timber.

Timber beams with ellipse-shaped cavities marked ME_5m-n, and normal (without cavities) marked MP_5m_n, were tested, where “n” represents the number of the tested specimen.

The linear behavior of the elements can be seen in the diagrams, after which a brittle fracture occurred. Furthermore, a minimal force reduction is observed in the diagrams, indicating the cracking of a particular lamella or grain. After that, the force increases again until the fracture of the next grain, and so on, until the complete fracture of the sample occurs when it reaches its tensile strength (Figure 6 and Figure 7).

The higher slope of the force-displacement curve for normal MP-n specimens can be seen in Figure 6, which indicates their greater stiffness, which is natural considering the perforation of the ME-n specimens, i.e., the smaller cross-sectional area.

A comparison of the results can be seen in Table 1 and Table 2. It can be concluded that the load-bearing capacity of the hollow GL timber specimens was reduced by 35% compared to the normal GL timber specimens.

In Figure 7, it can be seen that the failure of samples with the same type of cavities did not occur at the same load value. The cause was mostly natural wood defects, such as knots, shakes, cross-grain, crookedness, rind galls, burrs, and curls.

### 2.2. Fire Tests

This article contains the results of a fire test of glue-laminated normal and hollow timber beams in accordance with the procedures of the reference standards EN 1363-1:2020 [28] and HRN EN 1365-3:2002 [29].

**Figure 8:**
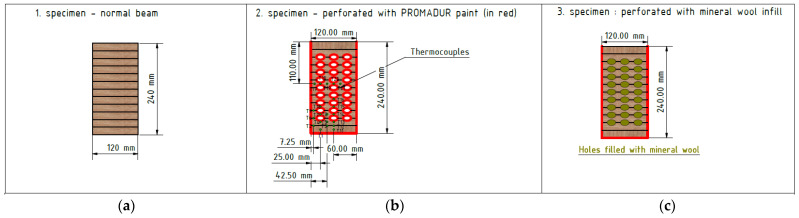
Specimen cross-section: (**a**) normal beam; (**b**) hollow with intumescent paint; (**c**) hollow with mineral wool infill.

**Figure 9:**
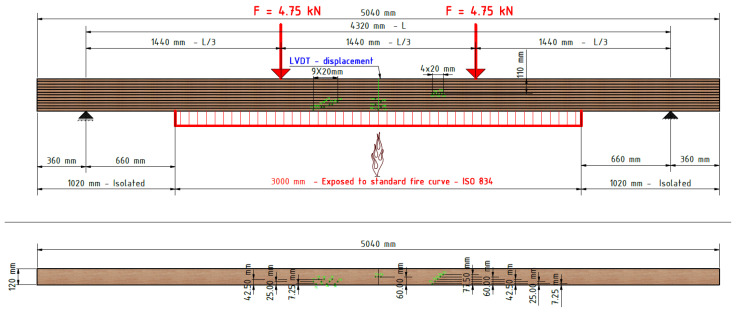
Fire test–experiment setup.

The dimensions of the samples were the same as in the mechanical tests, as follows: 120 × 240 × 5040 mm (width × height × length). Moisture was measured in each sample and the average moisture was 10% for all samples. The laminated timber sample consisted of a total of twelve lamellae glued together. The slats were made of soft timber (fir) 20 mm thick and made in such a way that the fibers inside the timber were directed in the direction of the length of the beam (see Figure 8 and Figure 9). The lamellas were glued using Kleiberit 510.0 glue, manufactured by Klebchemie-M.G. Becker GmbH [24]. This is a PUR adhesive for load-bearing wood construction certified in accordance with DIN 1052 [30]. An advantage and difference compared to previous research [23] was that this adhesive has a very high bond strength due to specially reinforced fibers. Three different types of timber beams were tested to compare innovative hollow glue-laminated timber elements with standard and most used timber products. Therefore, the first sample was a standard GL timber beam, and the other two were hollow GL beams with fire protection. In the first hollow beam, the protection was an intumescent paint coating, Promadur [31], along the perimeter of the beam and inside the cavity, while in the second the mineral wool was placed in cavities and intumescent paint was also applied on the outer surface of the sample. In this research, PROMADUR [31] intumescent paint was used. It is a single-pack water-borne, solvent-free transparent intumescent coating for fire protection of timber structures. In the case of fire, PROMADUR expands, creating a protective insulating foam that protects the substrate from contact with air (oxygen), decreasing the combustibility, and slowing down the transfer of energy (heat) from the fire to the timber elements, increasing the fire resistance. The general idea was to use cavities in such a way as to partially stop the penetration of fire and temperature towards the interior of the beam. Figure 8 shows different types of cross-sections of investigated timber beams.

The samples were exposed to fire on three sides (bottom and sides). The load-bearing beam specimen was mounted on a vertical load-bearing structure equipped with a hydraulic system for applying the load. The load-bearing structure was placed on the horizontal test furnace in such a way that only a part of the beam 3000 mm long was exposed to fire. The beam was oriented so that the longer side of the section (240 mm) was placed vertically. The beam supports were located outside the test furnace at a distance of 4320 mm from each other. On one side of the beam, one support was fixed, and on the other side, it was sliding in the longitudinal direction. All the above described can be seen in Figure 9.

The ceiling of the furnace was made using 150 mm-thick aerated concrete floor slabs. In the central part of the furnace, a 320 mm-wide opening was left between them, inside which a sample was installed. In this way, fire exposure from three sides of the sample was enabled. A 40 mm-thick calcium silicate plate of sufficient strength was placed on the upper surface of the sample to allow a load to be applied across it. The plate was of such width as to close the opening in the ceiling of the test furnace. The gap between the sample and the edges of the test furnace was closed with ceramic wool.

**Figure 10:**
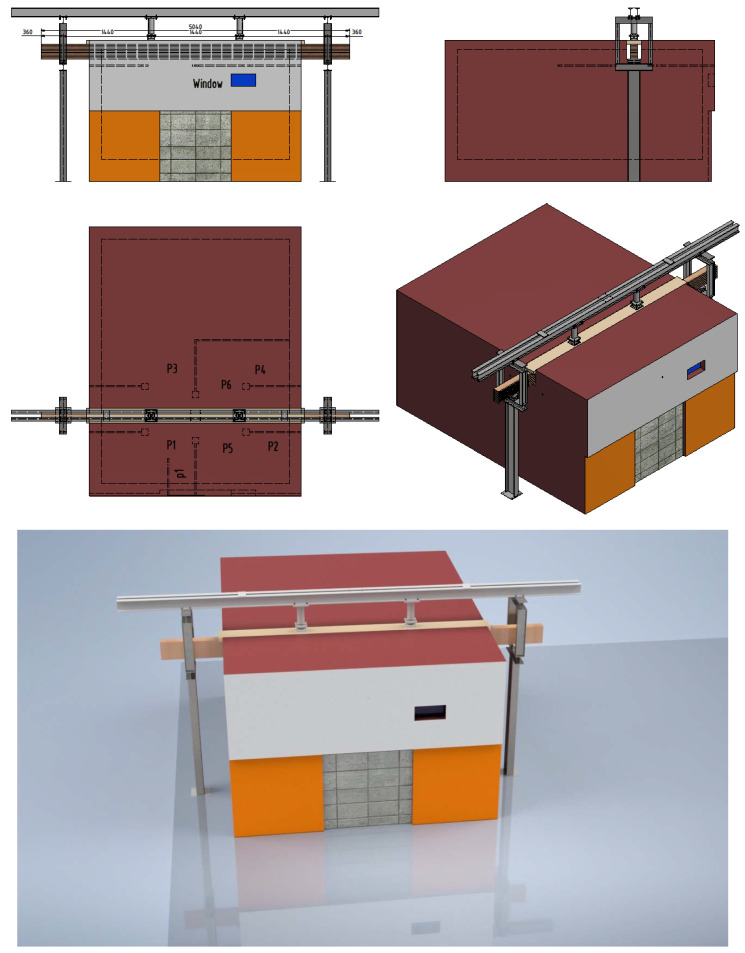
Furnace and general fire test scheme.

The construction, composition, and orientation of the sample as well as the type of sample supports on the load-bearing structure, are shown in Figure 10.

Prior to installation, the test sample was placed in a laboratory where the ambient conditions were maintained at approximately 50% relative humidity and 20 °C, in accordance with HRN EN 1363-1: 2020, clause 8.1 [28].

The percentage of moisture retained in the sample was measured immediately before the test and was 12.7%.

The fire furnace allowed standard exposure of test specimens to fire with respect to heat exposure and pressure. The fire in the fire area of the furnace was realized by means of six burners on liquid fuel (heating oil), in accordance with the standard HRN EN 1363-1: 2020, chapters 4.1 and 4.2 [28]. Air temperature in the test room 24 h before the fire test was maintained at 20 (±5) °C. Furnace heating was determined by a standard temperature curve, in accordance with the standard HRN EN 1363-1: 2020, 5.1 [28] and defined according to the following formula:

T = 345log(8t + 1) + 20(7)

where T is the average temperature in the furnace (°C), and t is time (min.)

The temperature in the furnace was measured with six thermocouples of type K, whose hot joint was fixed in the geometric center of the plate in accordance with the standard HRN EN 1363-1: 2020, 4.5.1.1 [28].

The thermocouples were evenly distributed along with the test sample and positioned so that they were not in contact with the open flame from the burner and were 100 mm away from the fire-exposed side of the test sample.

Static pre-pressure in the fire chamber of the test furnace was maintained in the range of 15 ± 3 Pa. The sensor-pressure gauge was placed so that the pressure was measured 100 mm below the level of the installed test sample and regulated by the closing part of the main and unloading chimney of the furnace.

**Figure 11:**
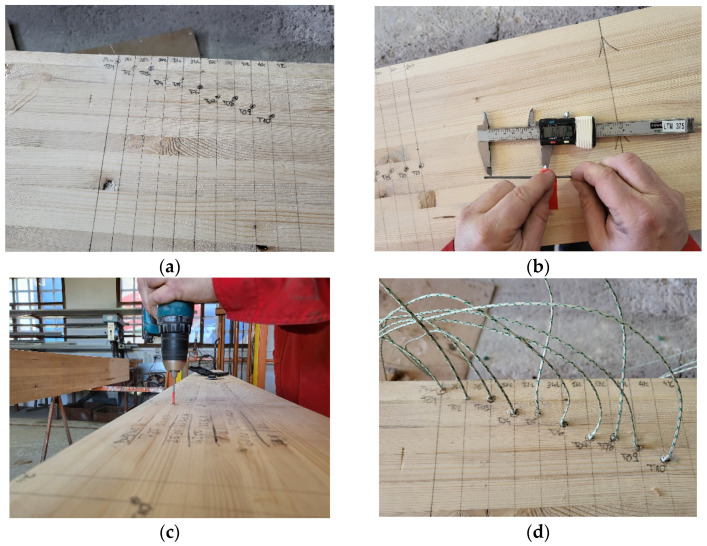
Thermocouple installation process: (**a**) marking the beam; (**b**) measuring and marking the drill; (**c**) hole drilling; (**d**) installation of the thermocouples.

Temperatures through the sample cross-section were measured with 21 NiCr-Ni thermocouples, (type K) wire Ø 0.5 mm. The installation process of thermocouples can be seen in Figure 11. After insertion, the thermocouples were additionally fixed with wooden wedges, while the holes were filled with fireproof silicone. In this way, the position and depth of the thermocouples were fully ensured.

**Figure 12:**
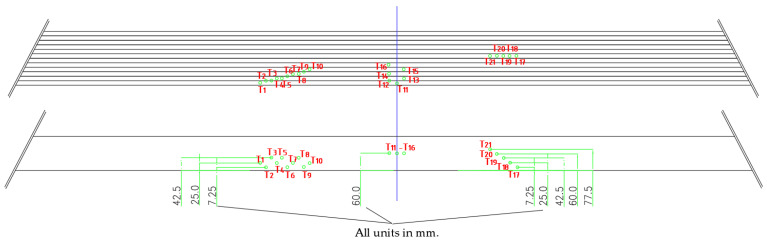
Thermocouple layout.

The thermocouple layout is shown in Table 3 and Figure 12, while thermocouple installation depth is shown it Table 4.

The layout of the thermoelements was chosen to cover temperature measurements from all exposed sides. The thermocouples were divided into three groups with seven thermocouples in each series depending on their placement in the beam. The group of thermoelements T11–T16 measured the development of temperature due to the influence of fire exposure from the bottom side, the group T17–T21 measured the temperatures assumed to mainly depend on the fire exposure from the side of the beam, while the group of thermoelements T1–T10 measured the notional charring rate and development of temperature.

In addition to temperature development, the deflection D_1_, in the middle of the beam was measured. To meet the sample load criteria, the deflection must be less than:

D_limit_ = L^2^/(400 · d) = 4320^2^/(400 × 240) = 194.4 mm (8)

Furthermore, the deflection increment rate must be less than:

(9) $${\left(\frac{\mathrm{dD}}{\mathrm{dt}}\right)}_{\mathrm{limit}}=\frac{L^{2}}{9000\;\cdot \;d}=\frac{4320^{2}}{9000\times 240}=8.64\;\mathrm{mm}/\min$$

The failure according to the load-bearing criteria occurred when the following conditions were met:

D_1_ ≥ 1.5 × D_limit_ ≥ 1.5 × 194.4 = 291.6 mm (10)

or

D_1_ > D_limit_ → D_1_ > 194.4 mm (11)

and

(dD_1_/dt) > (dD/dt) _limit_ → (dD_1_/dt) > 8.64 mm/min (12)

The load conditions were applied in accordance with the standard HRN EN 1365-3: 2002, point 7.3 [29]. The sample was loaded at two points, located on thirds of the length of the beam between the supports, with 6.5 kN, which corresponds to the total applied load on the sample in the amount of 13 kN for the normal beam, and 4.8 kN (in thirds), in total 9.6 kN for hollow beams.

The required load was achieved using a hydraulic system acting on the test specimen by means of two cylinders. Hydraulic cylinders with a maximum stroke of 100 mm were used, thus limiting the range of deflection of the sample at which the required load could be achieved.

The load was applied to the sample in the middle of the beam width at a distance of 1440 mm.

**Figure 13:**
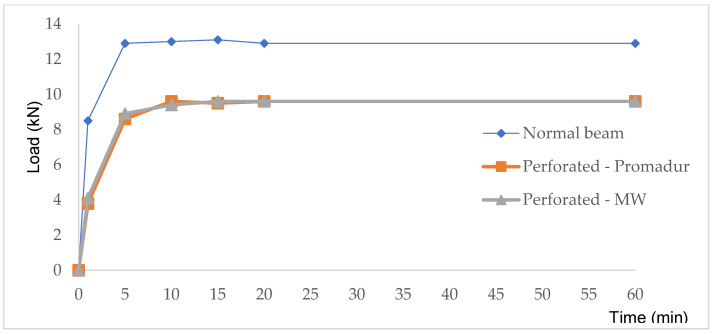
Loading protocol.

The sample was gradually loaded before the test. The required total load was reached 15 min before the start and was maintained throughout the test (see Figure 13).

## 3. Results

### 3.1. Normal Beam

The great advantage of this fire test is that a small window was made in the furnace through which the behavior of the beams could be observed and documented. Table 5 shows the sequence of major events during the fire test of a normal beam.

**Figure 14:**
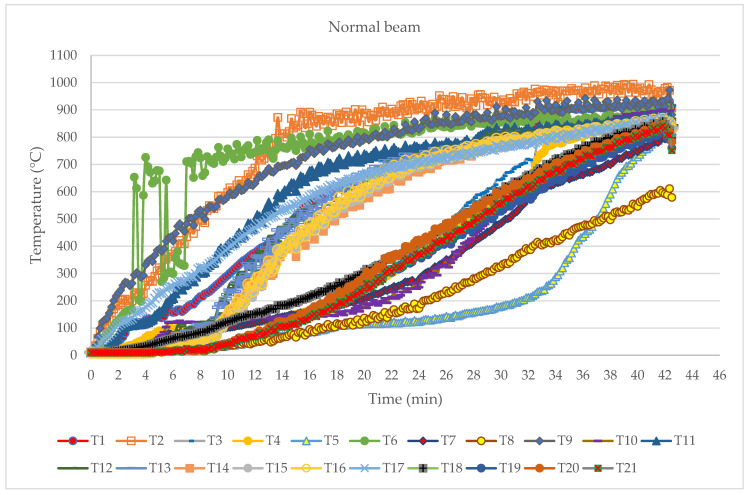
Temperature development diagram.

In addition to the observation of physical changes in the samples, the development of temperature towards the interior of the timber beams was monitored. The arrangement and selection of thermocouples made it possible to monitor the development of temperature for all three exposed sides of the samples. The following are the results of testing, i.e., the development of temperature over time (Figure 14).

The development of the temperature inside the sample was visible according to the groups of thermocouples. It could be seen that the group of thermocouples that was installed the deepest had a gentle slope of the curve, which told us about the natural resistance of wood as a material, i.e., the creation of a carbon layer. One important aspect was that all thermocouples in the thermocouple series were exposed to temperatures over 300 °C, which is where the isotherm charring line occurred. When thermocouples were directly exposed to fire they were affected by convection and radiation which can explain the temperature fluctuations in the diagrams (see T6 in Figure 14). From that point on, measurements of those thermocouples could be ignored.

**Figure 15:**
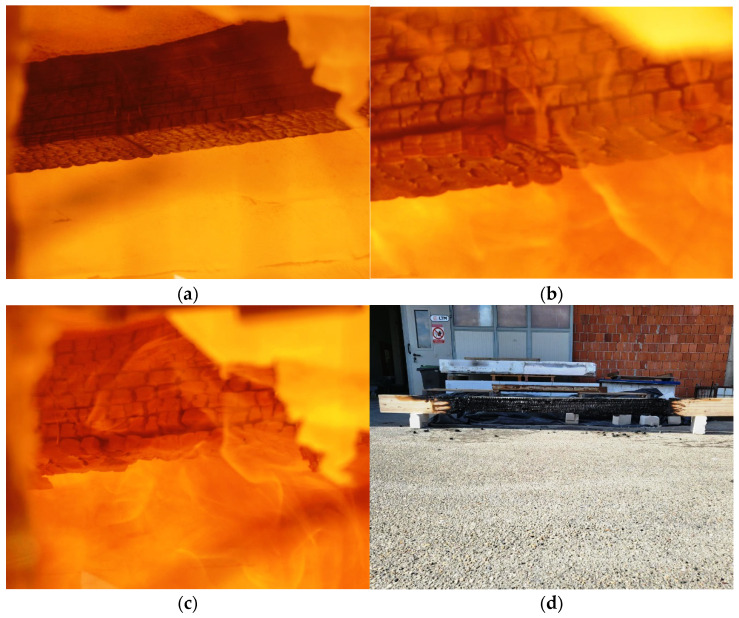
Normal beam—phases of the fire test: (**a**) 5 min; (**b**) 20 min; (**c**) 40 min; (**d**) the end of the test.

Different phases of normal GL timber beam in the fire tests are shown in Figure 15a–c, while burnt beam at the end of the test can be seen in Figure 15d.

### 3.2. Hollow Beam with Intumescent Paint—Promadur

**Figure 16:**
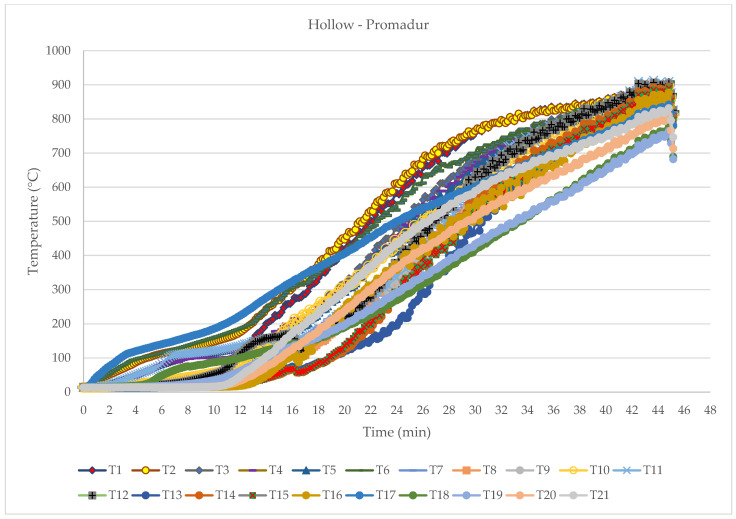
Temperature development diagram—hollow Promadur sample.

The main difference between normal beams and protected hollow beams was the slope of the temperature curves (Figure 16). These show the effect of intumescent paint, which protected samples for a certain time, which of course affected the final time of fire resistance of the samples. The general conclusion corresponds to the initial assumptions about the similar time of fire resistance of tested beams, which means that the fire protection fulfilled its purpose. The main observations during the fire tests can be seen in Table 6.

**Figure 17:**
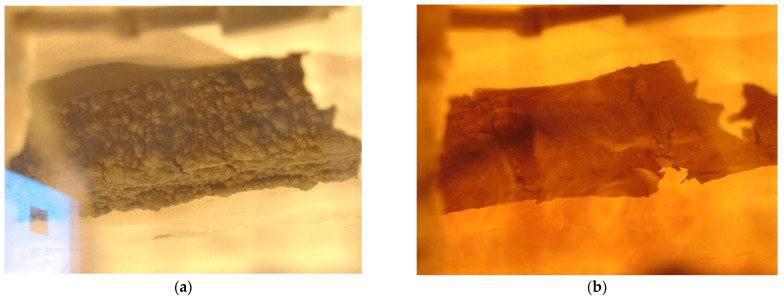

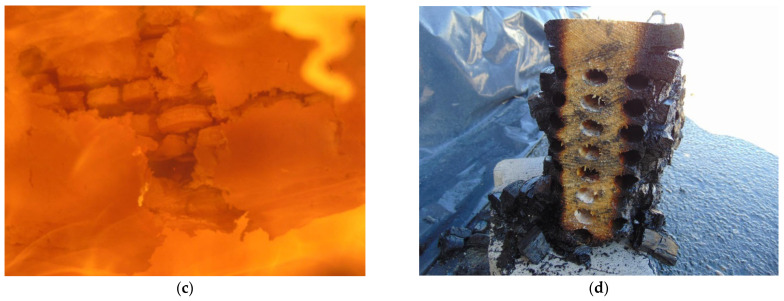
Hollow beam (Promadur)—phases of the fire test: (**a**) 5 min; (**b**) 20 min; (**c**) 41 min; (**d**) the end of the test.

Different phases of hollow (Promadur) GL timber beam in the fire tests are shown in Figure 17a–c, while burnt beam at the end of the test can be seen in Figure 17d.

### 3.3. Hollow Beam with Mineral Wool Infill

**Figure 18:**
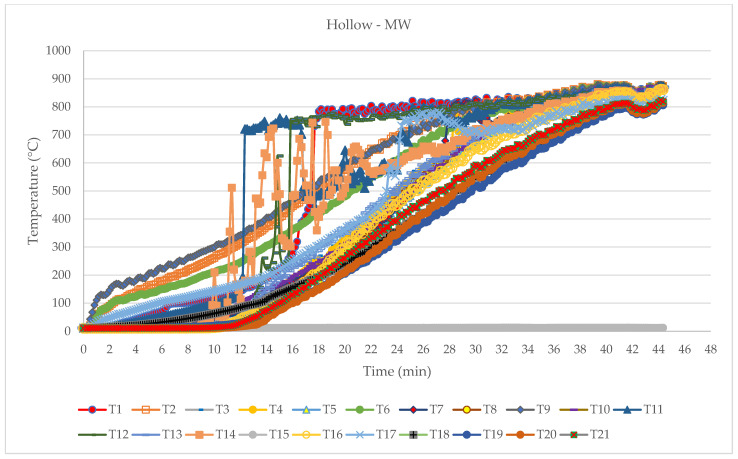
Temperature development diagram—hollow MW.

The sample with mineral wool infill behaved very similarly to the previous sample. The reason for this was that both samples had intumescent paint on the outside surface, and the difference was manifested in the fact that the mineral wool quickly fell off the sample and the fire reached the unexposed part of the timber sooner. This can also be seen in Figure 18, where we see more “lost” thermocouples that have fallen off the sample. The main observations during the fire test can be seen in Table 7.

**Figure 19:**
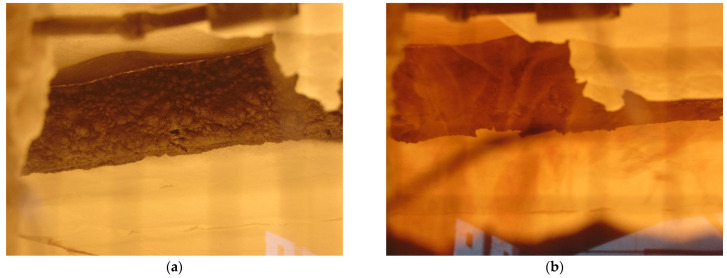

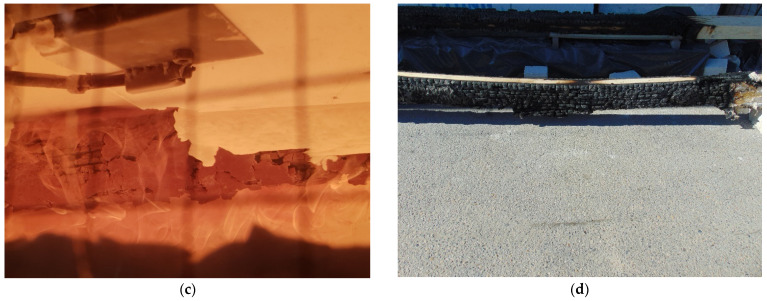
Hollow beam (Mineral wool)—phases of the fire test: (**a**) 5 min; (**b**) 20 min; (**c**) 41 min; (**d**) the end of the test.

Different phases of hollow (with MW) GL timber beam in the fire tests are shown in Figure 19a–c, while burnt beam at the end of the test can be seen in Figure 19d.

**Figure 20:**
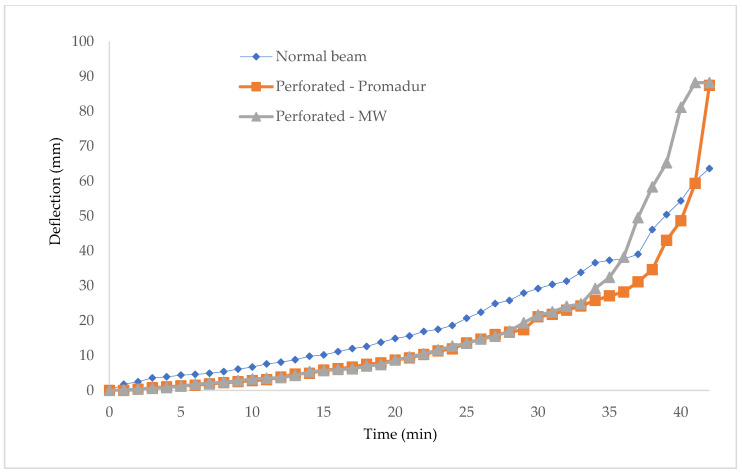
Deflection of the specimens in the midspan.

**Figure 21:**
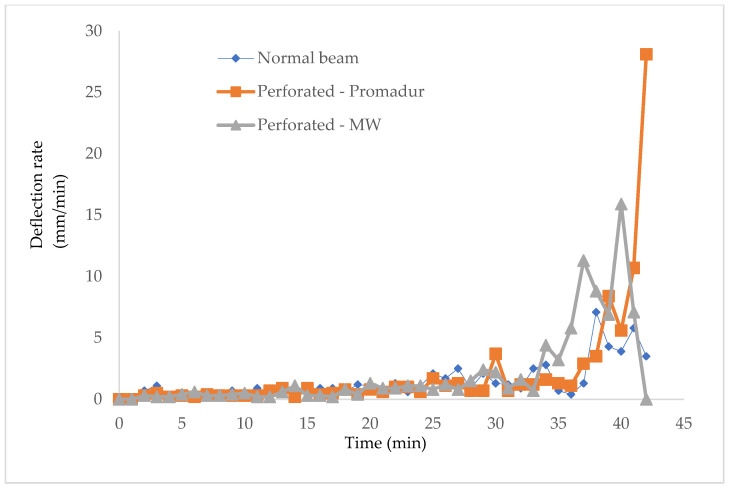
Deflection rate.

One of the main parameters for controlling the fire resistance of a beam is deflection. The deflection is conditioned and directly related to the burning of the beam and the development of temperature, as a physical consequence. Namely, during a fire event, a charred layer appears on the timber surfaces exposed to the fire, which protects the interior of the timber from heat development. Char depth is the distance from the outer edge of the element before the fire to the inner edge of healthy, unburnt timber, after the fire load. An increase in temperature leads to a decrease in material and geometric characteristics, which increases deflection. The size of the deflection and the rate of increase of the deflection can be seen below (Table 8, Figure 20 and Figure 21).

The tests were interrupted due to a flame penetration at the boundary between the exposed and non-exposed sides of the sample and a hazard to laboratory personnel and equipment.

### 3.4. Charring Behaviour

**Figure 22:**
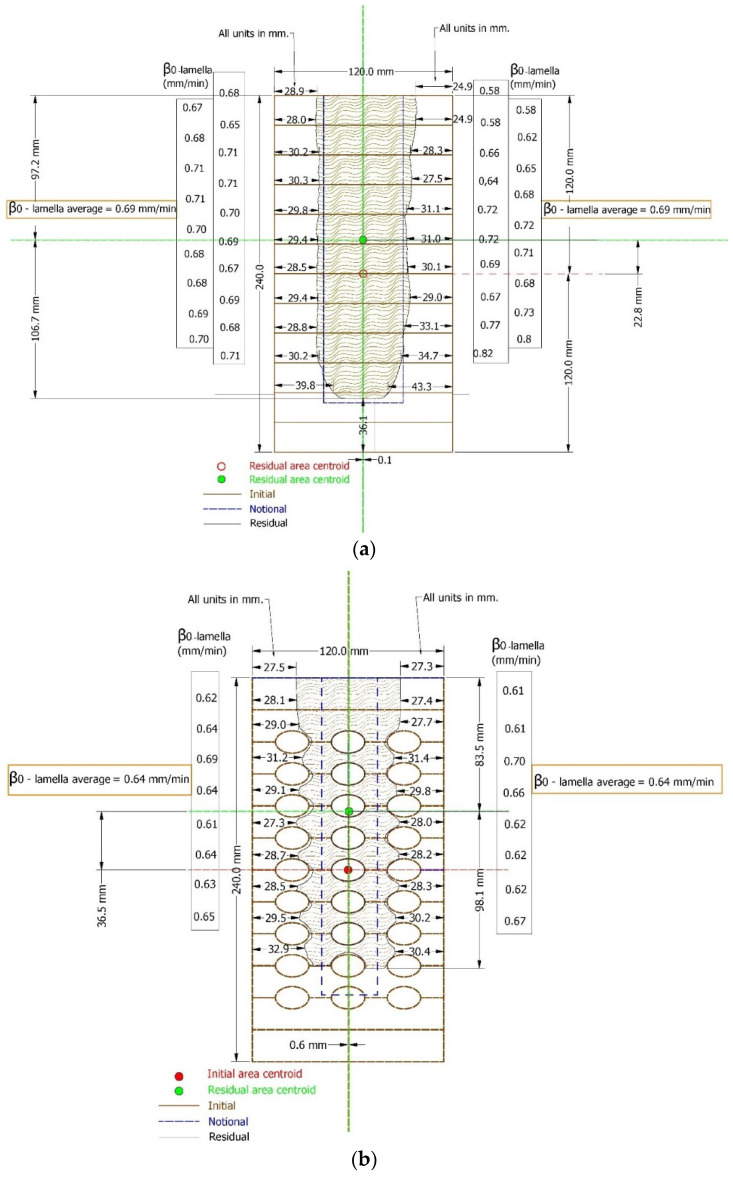
Residual cross-section analyzed with CAD software, dimensions in (mm): (**a**) normal GL beam; (**b**) hollow GL beam protected by intumescent paint.

The residual cross-section of the fire-exposed area of each beam was cut with equal spacing of 200 mm. After marking the cut parts, each part was scanned. The scanned files were then processed and imported into the CAD program for further analysis (Figure 22). In this way, the area, the moment of inertia, and the section moment of the residual cross-section were obtained. The last step was to determine the notional charring rate and notional section. Additionally, one-dimensional charring rates were measured for each lamella.

For a normal GL beam, the average one-dimensional charring rate β_0_ was 0.69 mm/min, while the notional charring rate was 0.78 mm/min. Due to fire protection and intumescent paint, for hollow GL beams the average one-dimensional charring rate β0 was 0.64 mm/min. When calculating the notional charring rate for hollow GL elements, a problem arose due to the existence of holes. When the notional charring rate was being measured, the section moment was considered, which was significantly lower for hollow GL beams (due to the presence of holes) compared to normal beams. Consequently, the notional charring rate would be quite a bit higher (0.95 mm/min). Therefore, the rule suggested by EN 1995-1-2 was used, which suggests that the ratio of one-dimensional charring rate to notional charring rate is 1.08, which results in 0.75 mm/min for a notional charring rate. Following Section 2.1 and Figure 3, the ideal residual cross-section is given by a blue dashed line in Figure 22.

## 4. Discussion

When designing timber structures in fire, EN1995-1-2: 2004 [25] provides basic guidelines and calculation methods (reduced cross-section and reduced strength and stiffness), which are based on testing with a standard fire curve. The resistance of GLT beams is usually determined according to given calculation methods or experimental testing. Evaluation of results testing is accompanied by uncertainty regarding the properties of the material, which, however, are necessary to know in order to obtain test results that could be compared with the reduced cross-section method according to EN 1995-1-2 [25]. Nevertheless, Schmidt [32] has shown the limitations of this study method. This primarily refers to the time of the beginning of charring, the rate of combustion, and the layer of zero strength. The difference in charring depth between model and fire testing increases with increasing fire exposure time. EN 1995-1-2 [25] prescribes the depth of the zero-layer strength of 7 mm for GLT beams. The basic information and limitations in determining the zero-strength layer have been well summarized by Schmid et al. [32,33]. Kamenicka et al. indicate the differences between simplified and advanced methods and give a comparison of the results [34]. It is convenient to use modeling in FE programs where it is possible to consider the change in thermal and mechanical properties of materials with increasing temperature in time. In the case of analytical methods, it is necessary to apply parameters that depend on the time of fire exposure [35,36]. According to the latest studies, the rate of timber burning in a natural fire may be higher than that in a standard fire curve. Due to all the above, a new Eurocode 5 (EN 1995-1-2: 2025) is currently being developed, which refers to the calculation of timber structures in a fire. Major additions and improvements relate to the sizing rules of CLT elements, timber–concrete composite systems, timber framework systems, joints, and details including adhesives, protection, and finally simplification and harmonization of calculation methods.

Innovative hollow wooden elements have certain advantages over normal, GL elements. Primarily, these relate to weight, assemblage, installation, and energy efficiency, but the subs themselves also bring certain potential disadvantages. First, problems can arise during a fire event. There are two key reasons for this: the reduced cross-section area and the possibility of oxygen passing through the cavities, which accelerates combustion. Furthermore, the reduced glued surface in the plane of the lamella joint can result in delamination. This is also the main reason for the earlier collapse of the hollow beams. In the case of normal timber beams, which have an adhesive surface over the entire width, delamination does not occur, but the beams fail due to the reduction of the cross-section. In contrast to solid beams, delamination occurred in the case of hollow beams because the glued surface is noticeably smaller. Considering the problem of delamination of the first exposed lamellas with hollow beams, in future research, the idea is to make the first lamellas without hollows and thus increase the bonding surface. Additionally, a higher quality of timber can be used for the first lamella and thus reduce the charring rate of timber.

**Figure 23:**
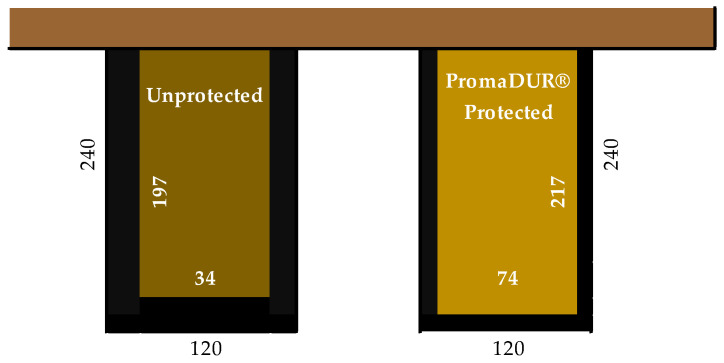
Comparison of the residual cross-section of unprotected and protected beams with Promadur after 45 min of fire exposure.

However, intumescent paint and mineral wool slowed down the penetration of the fire and charring, but the failure time was similar. Eurocode 5 (EC 1995-1-2) [25] provides procedures for calculating the fire resistance of structural timber elements with surfaces initially protected from fire exposure. The Eurocode 5 procedure divides the rated time periods into different intervals, with different charring rates, depending on the behavior of the protective material. As for the intumescent paint, PROMADUR coating was used. Fire rating of protected timber elements must be calculated based on the depth of char obtained from the value of t_f_ → t_ch_ (start of charring) and k_β_ (charring rate), from fire tests EN 13381-7 [37], as required by the Eurocode EC 1995-1-2 [25]. Considering the amount of coating applied (1120 kg/m^2^), the start of charring t_ch_ = 17 min was calculated, which was confirmed by the experiment. Furthermore, when the dimensions of the normal beam with and without PROMADUR were calculated and compared (Figure 23), the residual cross-section dimensions of the sample after exposure to fire for 45 min were 197/34 mm for the unprotected beam, and 217/74 for the protected beam. 

The mineral wool filling did not show a significant difference compared to PROMADUR. The reason for this is that there was no complete fire interruption inside the cross-section, along the entire width of the sample, which would completely prevent burning. Therefore, the non-combustibility of mineral wool cannot be fully manifested.

Finally, the limitations of this research are primarily the small number of specimens tested in a fire event. This makes it difficult to evaluate the influence of elevated temperature on the adhesive and the load-carrying capacity of glued-laminated hollow timber elements. The impact of the perforation of the timber element on the development of temperature towards the interior of the element was noticeable, but it cannot be concluded with certainty how much and in what way this affected the charring rate. In future research, the introduction of all mentioned problems in form of the analytical or numerical model is to be investigated.

EN 1995 prescribes the values of the notional charring rate β_n_ as well as the one-dimensional charring rate β_0_ for GL timber beams. However, innovative hollow GL timber elements are not applicable in recognized and current regulations such as EN 1995-1-2 [25], therefore further research and parametric analysis of numerous factors affecting fire resistance are required. Here, it can be concluded that special attention should be paid to how to introduce all the mentioned problems in form of the analytical and numerical model.

## 5. Conclusions

This study showed that the bond line behavior is dictated by both the adhesive performance and the adhesive-timber interaction. The behavior of different types of adhesives (PUR) at elevated temperatures used for the bond line between the lamellas had little influence on the fire resistance of the glued laminated timber elements. The reason for this lies in the fact that the adhesive line was generally too thin to have a great impact on heat development and finally, the fire resistance of timber elements. Furthermore, the adhesive decomposed at a temperature of about 300 degrees, and according to this experimental research, the mechanical properties (Strength, Young’s modulus, density) of timber approach zero at 300 degrees Celsius, so the impact of different types of adhesives is negligible. The one-dimensional charring rate for lamellae (β_0_ = 0.69 mm/min) was obtained, which is close to the value of 0.70 mm/min given in EN 1995-1-2. Furthermore, the notional charring rate for the beam (β_n_ = 0.78 mm/min) was presented for normal GL beams. In the case of hollow GL beams, due to the fire protection of intumescent paint there was a delay in the combustion of timber, which resulted in a lower one-dimensional charring rate (β_0_ = 0.64 mm/min). It can be concluded that in hollow GL elements the natural resistance of timber to fire (charred layer) and the prevention of heat propagation to the interior of the specimen, regardless of the existence of holes, were present. Consequently, the temperatures in the inner part of the hollow GL elements remained unchanged.

Heat propagation is independent of adhesive, which suggests that adhesive type did not enhance or prevent char propagation beyond the adhesive line. Comparing normal glue-laminated beams with hollow ones, the difference in failure modes could be seen, primarily because the appearance of debonding and delamination could be seen in the hollow elements. When delamination occurred, the char did not penetrate the full thickness of the first lamella. First, the reason for this is the reduced glued surface due to the perforation of the elements. When it comes to the specimen with mineral wool insulation inside the cavities, there was a positive effect on fire resistance, since there was no air in the cavities, but the non-flammable material retained the penetration of fire towards the bonding lines. Intumescent paint allowed a great improvement in the behavior and fire resistance of glue-laminated timber elements, thus prolonging the thermal wave to the adhesive line and consequently delamination. Delamination was observed from 100 to 300 °C. Using 130 °C as a design temperature might be over-conservative when compared to critical bond-line temperatures of 200 °C in large-scale tests. It is necessary to harmonize the methodology for testing the interaction of adhesive and timber in ambient and fire conditions.

In future research, a larger number of samples will be investigated for all types of GL timber elements, which will provide additional analysis of the behavior of GL timber elements in a fire event. This particularly applies to the hollow GL timber elements, where new additional parameters are included that influence the behavior of such elements in a fire event. Mainly, these refer to the geometry of the sample, i.e., the cavity, and occasionally, i.e., the air that circulates inside the holes.

Finally, the obtained results will enable an analytical and numerical analysis of the fire performance of GL timber elements.

## 6. Patents

The producer of the timber elements is a company (Tersa Ltd. from Croatia) that is in the application process for an intellectual property patent so that this product and system are protected.

## Figures and Tables

**Table 1:** Normal (non-perforated) samples—MP_5m results.

Specimen	Failure Load (kN)	Final Displacement (mm)	Moisture (%)
MP_5m_1	66.36	84.075	9.5
MP_5m_1	47.50	59.835	9.9
MP_5m_3	44.64	66.743	10.6
MP_5m_4	59.10	75.639	9.6
MP_5m_5	55.84	65.283	9.6
MP_5m_6	62.00	74.969	10.1
Average	55.90	71.09	9.93
Standard deviation	8.42	8.76	0.54
Variation coefficient	15.05%	12.32%	5.45%

**Table 2:** Perforated samples—MP_5m results.

Specimen	Failure Load (kN)	Final Displacement (mm)	Moisture (%)
ME_5m_1	31.48	54.625	11.2
ME_5m_2	46.60	71.522	9.6
ME_5m_3	29.53	51.443	10.2
ME_5m_4	39.05	61.094	9.9
ME_5m_5	28.85	52.289	9.2
Average	35.10	58.19	9.93
Standard deviation	7.60	8.35	0.59
Variation coefficient	21.65%	14.36%	5.99%

**Table 3:** Measuring points—thermocouple layout.

Measuring Points	Position
1, 11	mid-height of the 1st lamella
2, 3, 12	bondline—1st and 2nd lamellae
4, 5, 13	mid-height of the 2nd lamella
6	bondline—2nd and 3rd lamellae
7, 8, 14	mid-height of the 3rd lamella
9	bondline—3rd and 4th lamellae
10, 15	mid-height of the 4th lamella
16	mid-height of the 5th lamella

**Table 4:** Thermocouple installation depth.

Thermocouples	Depth Installation—Distance from the Exposed Side
2, 6, 9 and 17	7.25 mm
1, 4, 7, 10 and 18	25 mm
3, 5, 8 and 19	42.5 mm
11–16 m, and 20	60 mm
21	77.5 mm

**Table 5:** Observations during fire testing—normal beam.

Time (min:s)	Side (Exposed or Non-Exposed)	Observation
00:00		Start of the test
05:10	EX	The surface of the sample is completely charred, and flame occurs
12:20	EX	The lamellae at the bottom of the sample begin to separate—a gap of 3–5 mm.
27:10	EX	Fallen thermocouples were placed on the outside of the first lamella due to its combustion.
31:20	EX	Part of the lamellae of the first layer falls off.
36:10	EX	The next layer of lamellae in the middle of the sample is also separated.
42:20	NON-EX	A flame comes out on both sides of the sample between it and the insulation. The sample also burned outside the firebox area.
42:30		The end of the test.

**Table 6:** Observations during fire testing—hollow beam with intumescent paint.

Time (min:s)	Side (Exposed or Non-Exposed)	Observation
00:00		Start of the test
00:50	EX	The surface of the sample started to turn black (intumescent paint reaction).
01:30	EX	The intumescent paint completely blackened and began to swell.
14:20	EX	A visible opening in the layer of intumescent paint cover.
15:30	EX	A flame appears over the entire exposed surface of the sample.
17:10	NON-EX	Smoke comes out of the elliptical openings at the end of the beam.
35:20	EX	Parts of the swollen intumescent paint are falling off—most visible at the supports of the exposed beam
40:15	EX	Delamination is visible in the middle of the sample.
45:10		The end of the test.

**Table 7:** Observations during fire testing—hollow beam with mineral wool infill.

Time (min:s)	Side (Exposed or Non-Exposed)	Observation
00:00		Start of the test
00:50	EX	The surface of the sample started to turn black (intumescent paint reaction).
01:30	EX	The intumescent paint completely blackened and began to swell.
14:50	EX	A visible opening in the layer of intumescent paint cover.
16:50	EX	A flame appears over the entire exposed surface of the sample.
17:10	NON-EX	Smoke comes out of the elliptical openings at the end of the beam.
23:30	EX	Parts of the swollen intumescent paint are falling off—most visible at the supports of the exposed beam
39:50	EX	Mineral wool falling off and delamination are visible in the middle of the sample.
44:20		The end of the test.

**Table 8:** Deflection and deflection rate.

Sample	Deflection Rate—dD/dt Limit (mm/min)	Final Deflection—D1 (mm)	Test Stop (min)
Normal beam	7.1	63.6	43.
Hollow-Promadur	10.7	87.4	46.
Hollow-MW	15.9	88.2.	45.

## Data Availability

Data available on request due to restrictions, e.g., privacy or ethics. The data presented in this study are available on request from the corresponding author.
